# Machine learning based imputation techniques for estimating phylogenetic trees from incomplete distance matrices

**DOI:** 10.1186/s12864-020-06892-5

**Published:** 2020-07-20

**Authors:** Ananya Bhattacharjee, Md. Shamsuzzoha Bayzid

**Affiliations:** 1 Department of Computer Science and Engineering, Bangladesh University of Engineering and Technology, Dhaka, 1205 Bangladesh; 2 Department of Computer Science and Engineering, Eastern University, Dhaka, Bangladesh

**Keywords:** Phylogenetic trees, Species trees, Gene trees, Missing data, Imputation, Deep learning, Matrix factorization, Autoencoder

## Abstract

**Background:**

With the rapid growth rate of newly sequenced genomes, species tree inference from genes sampled throughout the whole genome has become a basic task in comparative and evolutionary biology. However, substantial challenges remain in leveraging these large scale molecular data. One of the foremost challenges is to develop efficient methods that can handle missing data. Popular distance-based methods, such as NJ (neighbor joining) and UPGMA (unweighted pair group method with arithmetic mean) require complete distance matrices without any missing data.

**Results:**

We introduce two highly accurate machine learning based distance imputation techniques. These methods are based on matrix factorization and *autoencoder* based deep learning architectures. We evaluated these two methods on a collection of simulated and biological datasets. Experimental results suggest that our proposed methods match or improve upon the best alternate distance imputation techniques. Moreover, these methods are scalable to large datasets with hundreds of taxa, and can handle a substantial amount of missing data.

**Conclusions:**

This study shows, for the first time, the power and feasibility of applying deep learning techniques for imputing distance matrices. Thus, this study advances the state-of-the-art in phylogenetic tree construction in the presence of missing data. The proposed methods are available in open source form at https://github.com/Ananya-Bhattacharjee/ImputeDistances.

## Background

Phylogenetic trees, also known as evolutionary trees, represent the evolutionary history of a group of entities (i.e., species, genes, etc.). Phylogenetic trees provide insights into basic biology, including how life evolved, the mechanisms of evolution and how it modifies function and structure. One of the ambitious goals of modern science is to construct the “Tree of Life” – the evolutionary relationships among all the organisms on earth. Central to assembling the tree of life is the ability to efficiently analyze a vast amount of genomic data.

The field of phylogenetics has experienced tremendous advancements over the last few decades. Sophisticated and highly accurate statistical methods for reconstructing *gene trees* and *species trees* are mostly based on maximum likelihood or Markov Chain Monte Carlo (MCMC) methods, and probabilistic models of sequence evolution (see [1] for example). Various coalescent-based species tree methods – with statistical guarantees of returning the true tree with high probability given a sufficiently large number of estimated gene trees that are error-free – have been developed, and are increasingly popular [2–12]. However, many of these methods are not scalable to analyze phylogenomic datasets that contain hundreds or thousands of genes and taxa [13, 14]. Therefore, developing fast yet reasonably accurate methods is one of the foremost challenges in large-scale phylogenomic analyses. Distance-based methods represent an attractive class of methods for large-scale analyses due to their computational efficiency. Although these methods are generally not as accurate as the computationally demanding Bayesian or likelihood based methods, several studies [10, 11, 15–19] have provided support for the ability of the distance-based methods in estimating accurate phylogenetic trees. Therefore, the trees estimated by distance-based methods can be used as *guide trees* (also known as *starting trees*) for other sophisticated methods as well as for divide-and-conquer based boosting methods [14, 20–24]. Moreover, under various challenging model conditions, distance-based methods become the only viable option for constructing phylogenetic trees. Whole genome sequences are one such case, where the traditional approach of multiple sequence alignments may not work [25]. Auch et al. (2006) proposed a distance-based method to infer phylogeny from whole genome sequences and discussed the potential risks associated with other approaches [26]. Gao et al. (2007) also introduced a composite vector approach for whole genome data, where distances are computed based on the frequency of appearance of overlapping oligopeptides [27]. Therefore, notable progress has been made towards developing various distance-based methods [1, 10, 11, 16, 17, 19, 28–35]. Some of these methods can also be used to analyze large-scale single nucleotide polymorphism (SNP) data [36, 37].

Missing data is considered as one of the biggest challenges in phylogenomics [38–40]. Missing data can arise from a combination of reasons, including data generation protocols, failure of an experimental assay, approaches to taxon and gene sampling, and gene birth and loss [36, 41]. The presence of taxa comprising a substantial amount of missing (unknown) nucleotides may significantly deteriorate the accuracy of the phylogenetic analysis [40, 42, 43], and can affect branch length estimations in traditional Bayesian methods [44]. Therefore, many studies avoid working with missing data and conduct experiments on the available complete dataset [39]. Several paleontology-oriented studies also suggest that missing data can frequently result in poorly resolved phylogenetic relationships [42, 45, 46].

Several widely used distance-based methods, including NJ [16], UPGMA [28], and BioNJ [17] require that the distance matrices do not contain any missing entries. However, only a few studies have addressed the problem of imputing distance values [36, 47]. These works mainly rely on two approaches, namely *direct approach* and *indirect approach*. Direct approaches try to construct a tree directly from a partially filled distance matrix [1, 48]. Indirect approaches, on the other hand, estimate the missing entries, and subsequently construct a phylogenetic tree based on the complete distance matrix [49, 50]. Some studies have tried to combine the advantages of both approaches [43]. LASSO [36], which is a heuristic approach for reconstructing phylogenetic trees from distance matrices with missing values, tries to exploit the redundancy in a distance matrix. This method, requiring the molecular clock assumption (i.e., sequences evolve at a constant rate over time and among different organisms [51, 52]), has been shown to be relatively less accurate by Xia et al. (2018), as significant differences were observed between the original trees and the trees reconstructed by LASSO from incomplete distance matrices [47]. Xia et al. (2018) proposed a least square method with multivariate optimization, which achieved a high accuracy for estimating trees from distance matrices with missing entries [47].

In this paper, we propose two statistical and machine learning (ML) based methods for imputing missing values in distant matrices. These methods do not require any particular assumptions (e.g., molecular clock) and can handle large numbers of missing entries. Our techniques are based on *matrix factorization* (MF) [53] and *autoencoders* (AE) [54]. We assessed the performance of MF and AE on a collection of real biological and simulated datasets. MF and AE were compared with the methods proposed by Xia et al. (2018) [47] (implemented in the DAMBE software package [55, 56]) and Kettleborough et al. (2015) [36] (implemented in the LASSO software package [57]). Experimental results suggest that MF and AE are more accurate and robust than DAMBE and LASSO under various model conditions, and can handle large numbers of missing values.

## Results

We compared our methods (MF and AE) with two of the most accurate existing methods: 1) DAMBE (the imputation method proposed by Xia et al. (2018) [47], and 2) LASSO [36]. We used a collection of previously studied simulated and biological datasets to evaluate the performance of these methods. We compared the estimated species trees to the model species tree (for the simulated datasets) or to the trees estimated on the full data without any missing entries (for the biological datasets), to evaluate the accuracy of various imputation techniques. We have used normalized Robinson-Foulds (RF) distance [58] to measure the tree error. The RF distance between two trees is the sum of the bipartitions (splits) induced by one tree but not by the other, and vice versa. Normalized RF distance (RF rate) is obtained by dividing the RF distance by the maximum possible RF distance. This error rate accounts for the number of different bipartitions between the inferred and the true phylogenies, and hence relatively lower error rates indicate better performance.

Similar to previous studies [47], we generated missing entries in two ways: i) modifying the input sequences in a way that results in missing entries in the distance matrix (indirect approach), and ii) directly deleting entries from a given distance matrix (direct approach). There are $\frac {n(n-1)}{2}$ distance values in a complete distance matrix of *n* taxa since the distance matrix is symmetric. For the direct approach, similar to previous studies [36, 47], we randomly removed some entries to create partial distance matrices. See the “Datasets” section for details on the indirect approach. We computed distances from the sequences based on the MLCompositeTN93 (TN93) model [59]. TN93 model holds the assumption of a complex but specific model of nucleotide substitution. The distance formula is derived under the homogeneity assumption, which means that the pattern of nucleotide substitution has not changed in the evolutionary history of the observed sequences [60, 61]. TN93 model accounts for the difference between transitional substitution rates, i.e., interchange of a purine nucleotide to another purine (*A*⇔*G*), or a pyrimidine nucleotide to another pyrimidine (*C*⇔*T*), and transversions (interchange of a single purine to a pyrimidine, or vice versa). TN93 also differentiates the two kinds of transitions (*A*⇔*G* and *C*⇔*T*). In addition to the TN93 model used in previous studies [47], we also applied the LogDet method [62] to observe its impact on the imputation process. The LogDet distance *d*_*xy*_ between two taxa *x* and *y* is defined as follows. Let *F*_*xy*_ be a *K*×*K* (*K*=4 for nucleotide sequences and *K*=20 for amino acid sequences) divergence matrix where *ij*-th entry is the proportion of sites in which taxa *x* and *y* have character states *i* and *j*, respectively. Then, *d*_*xy*_ is calculated using the following transformation [62, 63].

1 $$  d_{xy} = - \ln{[det F_{xy}]}.  $$

**Fig. 1:**
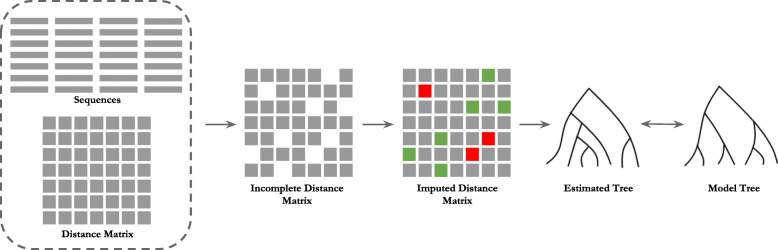
An overview of the experimental pipeline of this study. The input is either a set of sequences, or a complete distance matrix. We generate incomplete distance matrix from input sequences or input complete distance matrix by using various missingness mechanisms. Next, we apply various imputation techniques to impute the missing entries in the incomplete distance matrix and thereby, generating (complete) imputed distance matrices. Next, we estimate phylogenetic trees from the imputed distance matrices using FastME. Finally, we compare the estimated trees with the model tree to evaluate the performance of various imputation techniques

We used MEGA-X [61, 64, 65] to compute distances under the TN93 and LogDet models as well as to introduce missing entries in the distance matrices. We used FastME [19, 30] to construct trees from complete distance matrices. A schematic diagram of the experimental pipeline used in this study is shown in Fig. 1.

### Datasets

We have used a set of mitochondrial COI and CytB sequences from 10 Hawaiian katydid species in the genus *Banza* along with four outgroup species. This dataset, comprising 24 operational taxonomic units (OTUs) and 10 genes which evolved under the HKY85 model [66], was previously studied in [47]. In order to evaluate the relative performance, we followed exactly the same process used by Xia et al. (2018) [47] for modifying the sequences to create missing entries in distance matrices. However, Xia et al. (2018) only generated 30 missing entries in the matrix, whereas we analyzed a wide range of missing entries (10 ∼140).

We now explain how missing values were introduced by modifying the sequences. The 24 OTUs dataset comprises a set of mitochondrial COI and CytB sequences. If we remove the COI sequence from a taxon *A* and the CytB sequence from another taxon *B*, then the (*A*,*B*) pair does not share any homologous sites which results in a missing entry in the corresponding distance matrix. Thus, if we remove the COI sequence from *n*_1_ taxa and remove the CytB sequence from a different set of *n*_2_ taxa, the corresponding distance matrix will have *n*_1_×*n*_2_ missing entries.

We used another set of simulated datasets based on a biological dataset (37-taxon mammalian dataset [67]) which was generated and subsequently analyzed in prior studies [9, 14, 68, 69]. This dataset was generated under the multi-species coalescent model [70] with various model conditions reflecting varying amounts of gene tree discordance resulting from the incomplete lineage sorting (ILS) [71]. This collection of datasets was simulated by taking the species tree estimated by MP-EST [7] on the biological dataset studied in Song et al. (2012) [67]. This species tree had branch lengths in coalescent units that were scaled (multiplying or dividing by two) to vary the amount of ILS (shorter branch lengths result in more ILS). The basic model condition with moderate amount of ILS is referred to as 1X and the model conditions with higher and lower amounts of ILS are denoted by 0.5X and 2X, respectively. For each model condition, we used 10 replicates of data each containing 37 sequences. We analyzed a wide range of missing entries: 36 (6×6), 100 (10×10), 225 (15×15), and 342 (19×18).

In order to evaluate the performance of various methods on relatively larger datasets, we used a dataset containing 201 taxa, which was simulated and used by [72]. We analyzed various numbers of missing entries: 400 (20 ×20), 1,024 (32 ×32), 2,500 (50 ×50), 5,625 (75 ×75), and 10,100 (101 ×100).

We also analyzed three distance matrices, which were computed from aligned sequences from Carnivores, Baculovirus, and mtDNAPri3F84SE, and were used in previous studies [73, 74]. The numbers of taxa in these matrices range from 7 to 10. Various numbers of distance values were randomly removed to introduce missing data.

For each model condition with a particular number of missing entries, we generated 10 replicates of data, and reported the average RF rate and standard error over 10 replicates. However, we deliberately analyzed one replicate of data on 24 OTUs dataset as was done in Xia et al. (2018) [47] and removed the same entries that were removed by [47] to compare the performance of our proposed techniques with respect to the results reported in [47].

### Results on sequence input

Table 1 shows the results on 24 OTUs for a wide range of missing entries (10 ∼140). On this particular dataset, MF achieved superior performance on small to moderate numbers of missing entries (0 ∼40), LASSO matched or improved upon the other methods for moderate to high numbers of missing entries (50 ∼110), and AE outperformed other methods in the presence of large numbers of missing values (110 ∼140).

**Table 1:** RF rates of different methods on the 24 taxa dataset with varying numbers of missing entries. The best RF rates for various model conditions are shown in boldface

#Taxa	#Entries	#Missing	RF Rate			
		Entries	DAMBE	LASSO	MF	AE
		10	0.0476	0.2857	**0**	0.0952
		20	**0.1429**	0.3333	0.1905	0.2381
		30	0.2381	0.3333	**0.1905**	0.2381
		40	**0.2857**	0.3333	**0.2857**	0.3333
		50	0.3333	0.1905	0.4286	0.3333
		60	0.2857	**0.2381**	0.3333	0.381
		70	0.4286	**0.2857**	0.5714	0.381
24	276	80	0.4762	**0.381**	0.6667	**0.381**
		90	0.5714	**0.5238**	**0.5238**	**0.5238**
		100	0.5714	**0.5714**	0.7143	0.6190
		110	0.7143	**0.6190**	0.8571	**0.6190**
		120	0.8095	0.7619	0.8571	**0.7143**
		130	0.8571	**0.7619**	0.8095	**0.7619**
		140	N/A	N/A	**0.7619**	**0.7619**

**Fig. 2:**
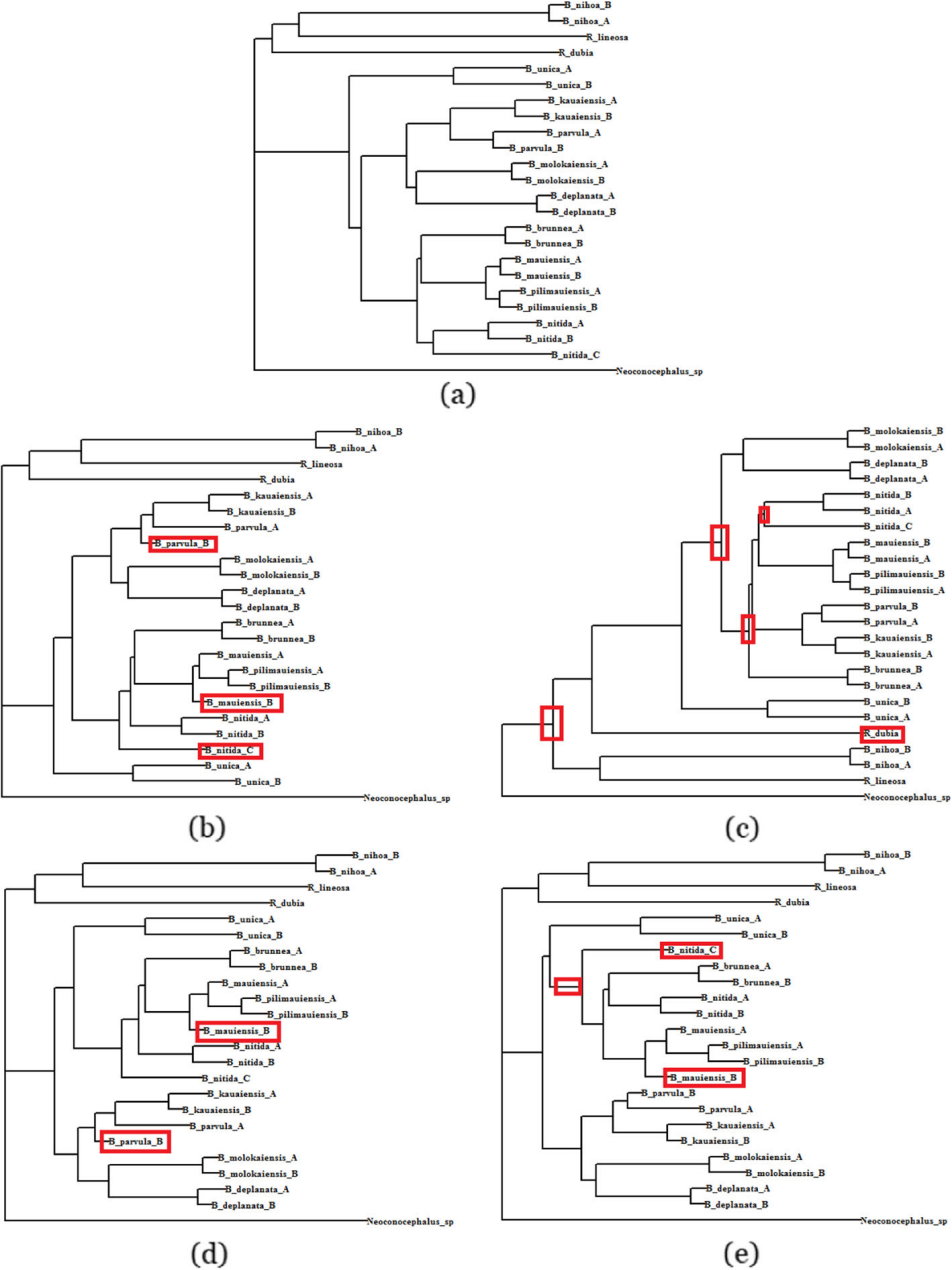
Phylogenetic trees estimated on the full and incomplete dataset (30 missing entries) with 24 OTUs from 10 Hawaiian katydid species. **a** Tree estimated from the full data (complete distance matrix), **b** - **e** trees reconstructed from incomplete distance matrix by DAMBE, LASSO, MF, and AE, respectively. Red rectangles highlight the inconsistencies with the tree on the full dataset

For 30 missing entries (which was the case analyzed in [47]), MF recovered 81% of the true bipartitions, whereas DAMBE and LASSO recovered 76% and 67% bipartitions respectively. Figure 2 shows the trees constructed by various methods with 30 missing values. With 10 ∼40 missing entries, MF estimated tree was closer to the tree estimated on the complete dataset than DAMBE and AE. Notably, with 10 missing entries, MF was able to reconstruct the tree on complete dataset, whereas DAMBE and AE incurred 5% and 10% errors, respectively. However, as we increase the number of missing entries, DAMBE started to outperform MF, and AE started to outperform both DAMBE and MF. Moreover, with moderate to high numbers of missing values (50 ∼110), LASSO achieved the best performance in recovering true bipartitions, although MF and AE were equally good in some cases. When around one-third (90) of the entries in the distance matrix were missing, LASSO, MF, and AE recovered around 48% of the true bipartitions, and DAMBE recovered 43% of the bipartitions. DAMBE can not impute distances when more than 50% of the total entries are missing. LASSO’s performance on these model conditions with a relatively large amount of missing data is also not satisfactory, since LASSO failed to construct a tree on the full set of taxa, resulting in an incomplete tree. Therefore, we did not consider DAMBE and LASSO when more than 50% of the entries (i.e., 140 entries on this particular dataset) are missing. On the other hand, both MF and AE were able to reconstruct around 25% of the true bipartitions even when 140 (more than 50%) entries are missing. Although, more than 50% missing entries in a distance matrix may not be a very common model condition, the ability to handle arbitrarily large amounts of missing data advances the state-of-the-art in distance matrix imputation.

Results on 37-taxon simulated dataset with varying amounts of ILS, two different evolution models and varying numbers of missing values are shown in Table 2. MF and AE were competitive with or better than DAMBE in most of the cases. Unlike the 24 OTUs dataset, LASSO performed poorly on this 37-taxon dataset, and achieved the worst tree accuracy. As DAMBE and LASSO can not handle distance matrices with more than 50% missing entries, only MF and AE were able to run on the distance matrices with 342 (∼50%) missing entries, albeit the RF rates were very high (due to the lack of sufficient phylogenetic information present in the highly incomplete distance matrix). MF could not recover any internal branches on the 1X dataset with 342 missing entries. AE, on the other hand, was able to reconstruct around 15% bipartitions. Another observation, within the scope of the experiments performed in this study, is that the amount of ILS does not have any significant impact on the performance of various imputation techniques. However, more experiments are required to further investigate the impact of ILS.

**Table 2:** Average RF rates (± standard error) of different methods on the 37-taxon dataset for varying numbers of missing entries and two different sequence evolution models. For each model condition, we show the average RF rate and standard error over 10 replicates. The best RF rates for various model conditions are shown in boldface

#Taxa	#Entries	Scaling	Model	#Missing	Average RF Rate			
				Entries	DAMBE	LASSO	MF	AE
				36	0.41 ±0.02	0.72 ±0.03	**0.33** ±0.03	0.41 ±0.02
				100	0.48 ±0.02	0.72 ±0.03	0.46 ±0.02	**0.45** ±0.02
			TN93	225	0.72 ±0.03	0.78 ±0.03	**0.62** ±0.01	0.70 ±0.02
37	666	1X		342	N/A	N/A	0.99 ±0.02	**0.86** ±0.01
				36	0.41 ±0.02	0.71 ±0.02	**0.35** ±0.03	0.4 ±0.02
				100	0.49 ±0.02	0.72 ±0.02	0.5 ±0.03	**0.46** ±0.02
			LogDet	225	0.72 ±0.02	0.76 ±0.02	**0.66** ±0.03	0.72 ±0.02
				342	N/A	N/A	1 ±0	**0.86** ±0.02
				36	0.45 ±0.02	0.69 ±0.02	**0.35** ±0.02	0.43 ±0.02
				100	**0.49** ±0.03	0.72 ±0.02	0.5 ±0.02	0.54 ±0.03
			TN93	225	0.66 ±0.02	0.76 ±0.02	**0.62** ±0.01	0.71 ±0.02
37	666	0.5X		342	N/A	N/A	1 ±0	**0.84** ±0.02
				36	0.45 ±0.02	0.68 ±0.02	**0.35** ±0.02	0.42 ±0.02
				100	**0.49** ±0.03	0.71 ±0.02	0.52 ±0.02	0.51 ±0.02
			LogDet	225	**0.64** ±0.02	0.76 ±0.01	0.66 ±0.02	0.7 ±0.02
				342	N/A	N/A	0.99 ±0.02	**0.84** ±0.02
				36	0.43 ±0.02	0.68 ±0.01	**0.36** ±0.03	0.42 ±0.02
				100	**0.5** ±0.01	0.69 ±0.02	0.52 ±0.02	**0.5** ±0.02
			TN93	225	**0.66** ±0.02	0.73 ±0.02	0.71 ±0.02	0.69 ±0.02
37	666	2X		342	N/A	N/A	0.99 ±0.01	**0.85** ±0.01
				36	0.44 ±0.02	0.63 ±0.02	**0.36** ±0.02	0.4 ±0.01
				100	**0.51** ±0.02	0.66 ±0.02	0.54 ±0.02	0.52 ±0.02
			LogDet	225	**0.66** ±0.02	0.73 ±0.01	0.7 ±0.02	0.69 ±0.02
				342	N/A	N/A	0.99 ±0.01	**0.86** ±0.02

We also analyzed the impact of two widely used sequence evolution models (TN93 and LogDet) on the performance of the proposed imputation techniques. MF performed poorly on LogDet model compared to the TN93 model, and produced higher error rates in 17 (out of 24) cases on LogDet model than the TN93 model. AE, on the other hand, showed similar (on 1X model) or slightly better (on 0.5X and 2X models) performance under the LogDet model. DAMBE achieved an improved performance under the LogDet model (compared to the TN93 model) only on the 0.5X model condition and the opposite trend is observed on the 1X and 2X model conditions, albeit the differences are very small (Table 2). LASSO performed slightly better on LogDet model than on TN93.

Finally, we applied our techniques on a large dataset with 201 taxa (Table 3). As DAMBE was too computationally expensive to run on this large dataset (it did not provide any result after 24 hours of computation), we excluded DAMBE from this analysis. Both MF and AE outperformed LASSO in all cases, and the improvements are substantial. AE performed particularly well on this dataset, as it achieved the lowest average RF rates under all model conditions. MF also performed well, and achieved comparable accuracies. The improvement of AE over MF and LASSO increases as we increase the number of missing entries. Even with 10,100 (∼50%) missing entries, AE was able to recover 57% true bipartitions under both sequence evolution models. LASSO consistently achieved the highest average RF rate under various model conditions. Even with only 400 (∼2%) missing entries, LASSO could not recover more than 41% true bipartitions, which is worse than AE’s performance on a model condition with 10,100 (∼50%) missing entries.

**Table 3:** Average RF rates (± standard error) of different methods on the 201-taxon dataset. The best RF rates for various model conditions are shown in boldface

#Taxa	#Entries	Model	#Missing	Average RF Rate		
			Entries	LASSO	MF	AE
			400	0.6 ±0.02	**0.36** ±0.04	**0.36** ±0.01
			1024	0.61 ±0.02	0.4 ±0.05	**0.39** ±0.04
		TN93	2500	0.62 ±0.02	0.41 ±0.03	**0.4** ±0.02
			5625	0.63 ±0.03	0.44 ±0.03	**0.41** ±0.03
201	20100		10100	N/A	0.59 ±0.02	**0.43** ±0.01
			400	0.59 ±0.02	0.38 ±0.02	**0.37** ±0.01
			1024	0.62 ±0.01	0.4 ±0.03	**0.38** ±0.02
		LogDet	2500	0.61 ±0.02	0.41 ±0.02	**0.4** ±0.02
			5625	0.62 ±0.02	0.46 ±0.03	**0.43** ±0.02
			10100	N/A	0.58 ±0.03	**0.43** ±0.01

### Results on distance matrix input

Results on Carnivores, Baculovirus and mtDNAPri3F84SE are shown in Tables 4, 5, and 6. On the Carnivores dataset (Table 4), LASSO and AE produced the best results. Even with 25 (more than 50%) missing entries, AE was able to reconstruct more than 25% of the true bipartitions. The performance of MF was worse than LASSO, AE, and DAMBE. On the Baculovirus dataset, MF achieved the lowest RF rates for relatively lower numbers of missing entries. However, as we increase the number of missing entries, LASSO and AE started to outperform other methods. On the mtDNAPri3F84SE dataset, these methods showed a mixed performance, and no method consistently outperformed the others. However, DAMBE and LASSO achieved better performance than MF and AE.

**Table 4:** Average RF rates (± standard error) of different methods on the Carnivores dataset. The best RF rates for various model conditions are shown in boldface

#Taxa	#Entries	#Missing	Average RF Rate			
		Entries	DAMBE	LASSO	MF	AE
		5	0.29 ±0.06	**0.14** ±0.06	0.37 ±0.1	0.23 ±0.07
		10	0.6 ±0.03	**0.23** ±0.07	0.71 ±0.06	**0.23** ±0.07
10	45	15	0.63 ±0.07	**0.26** ±0.02	0.83 ±0.09	0.57 ±0.04
		20	0.77 ±0.03	**0.4** ±0.06	0.94 ±0.07	0.63 ±0.05
		25	N/A	N/A	0.94 ±0.05	**0.74** ±0.05

**Table 5:** Average RF rates (± standard error) of different methods on the Baculovirus dataset. The best RF rates for various model conditions are shown in boldface

#Taxa	#Entries	#Missing	Average RF Rate			
		Entries	DAMBE	LASSO	MF	AE
		4	0.27 ±0.08	0.29 ±0.03	**0.17** ±0.15	0.39 ±0.04
		8	0.5 ±0.11	**0.33** ±0.08	0.5 ±0.1	0.39 ±0.08
9	36	12	0.7 ±0.07	**0.47** ±0.06	0.49 ±0.05	0.5 ±0
		16	0.7 ±0.06	**0.5** ±0.07	0.67 ±0.05	0.57 ±0.08
		20	N/A	N/A	**0.67** ±0.11	**0.67** ±0.11

**Table 6:** Average RF (± standard error) of different methods on the mtDNAPri3F84SE dataset. The best RF rates for various model conditions are shown in boldface

#Taxa	#Entries	#Missing	Average RF Rate			
		Entries	DAMBE	LASSO	MF	AE
		2	**0.05** ±0.05	0.1 ±0.04	0.4 ±0.15	0.15 ±0.09
		5	**0.2** ±0.08	**0.2** ±0.08	0.55 ±0.08	0.5 ±0.1
7	21	7	0.4 ±0.11	**0.3** ±0.13	0.75 ±0.07	0.8 ±0.19
		10	0.65 ±0.17	**0.5** ±0.16	0.8 ±0.04	0.7 ±0.04
		12	N/A	N/A	0.9 ±0.05	**0.85** ±0.05

### Running time

We performed the experiments on a computer with i5-3230M, 2.6 GHz CPU with 12 GB RAM. The running time of MF on the 24-taxon dataset ranges between 7 ∼15 minutes for various numbers of missing entries. DAMBE takes only a few seconds with 10 missing entries, but as we increase the number of missing entries to 130, the running time of DAMBE increases to 2 minutes. AE was faster, requiring only around 30 seconds for this dataset. LASSO was the fastest, which took only a second. Notably, unlike MF and DAMBE, the running times of LASSO and AE do not change much as we increase the number of missing entries.

On the 37-taxon dataset, MF takes around 30 minutes while DAMBE takes 12 ∼15 minutes. AE is faster than MF and DAMBE, taking only around 45 seconds. LASSO was the fastest method which took only a second. On 201-taxon dataset, DAMBE was too computationally expensive to run, and did not produce any result after 24 hours of computation. MF took 4 ∼6 hours, whereas AE took only 20 ∼30 minutes. LASSO was the fastest method which took only a second, although the accuracy was substantially worse than both MF and AE.

For relatively smaller matrices (Carnivores, Baculovirus and mtDNAPri3F84SE datasets), DAMBE is very fast, and finished in a second. MF took around 45 seconds, and AE took 20 seconds. Overall, AE and LASSO scale well to large datasets and their running times are less sensitive to the number of taxa and the number of missing entries.

## Discussion

We extensively evaluated MF and AE on a collection of real and simulated datasets. Previous studies [36, 47] limited their evaluation studies to a small number of datasets with limited numbers of taxa. Moreover, previous studies did not explore the model conditions with more than 10% missing values. We tried to address these issues by evaluating our methods on six different datasets with various challenging model conditions. We analyzed a 201-taxon dataset, whereas previous comparative studies were limited to less than 30 taxa. Furthermore, we analyzed the impact of varying amounts of ILS on the performance of various imputation techniques.

In general, MF and AE based methods produced more accurate trees than the existing methods. DAMBE was comparable to MF and AE when the numbers of missing entries were relatively small. However, DAMBE did not perform well with moderate to high numbers of missing entries. Although LASSO was previously shown to be less accurate than DAMBE [47], we found several cases where LASSO performed better than DAMBE. For relatively lower numbers of taxa, LASSO works very well, even when 25 ∼45% entries are missing. But on the 37-taxon and 201-taxon datasets, LASSO consistently performed poorly compared to other methods. On the other hand, MF and AE achieved superior tree accuracy on most of the model conditions. One prominent outcome of this study is the introduction of methods that can effectively analyze large datasets. While DAMBE failed to produce any results after 24 hours of computation and LASSO could not recover more than 40% true bipartitions on the 201-taxon dataset, the AE-based method consistently recovered around 60% bipartitions on this large dataset under various model conditions. Even the MF-based method, although less scalable than AE, showed promising performance, especially with relatively lower numbers of missing entries. The ability to analyze large datasets with hundreds of taxa makes our proposed methods applicable to large scale phylogenomic analyses.

Another important aspect is that both DAMBE and LASSO failed to handle distance matrices with more than 50% missing entries. However, MF and AE can handle an arbitrarily large amount of missing data. Sequence data may contain substantial amounts of missing information, resulting in distance matrices with large numbers of missing entries. We note that the presence of a substantial number of missing values in distance matrices may result in inaccurate trees and researchers will tend to approach these trees with care. However, the ability to construct trees in the presence of arbitrarily large numbers of missing entries will help us estimate starting trees (guide trees) on extremely challenging model conditions with high levels of missing data. These guide trees can be improved by further analysis (e.g., divide-and-conquer based boosting techniques [14, 20–23]).

Our extensive experimental studies on six different datasets suggest that AE-based method is more accurate and robust than others under most of the model conditions. Especially, on moderate to large-scale datasets and in the presence of relatively higher levels of missing data, AE is substantially better than the existing methods – making it a suitable candidate for large-scale phylogenomic analyses. This demonstrates the power of ML based techniques in capturing the latent representations in large-scale phylogenetic datasets, despite the presence of missing data. However, future works will need to investigate how to help the researchers choose the right imputation approaches on relatively small datasets as various methods have shown mixed performance on very small datasets (≤10 taxa).

Although we investigated a collection of datasets under various practical model conditions, this study can be extended in several directions. This study investigated relatively long sequences (250 ∼2600 bp); subsequent studies should investigate the relative performance of various methods on very short sequences. This study analyzed small to large scale dataset (7 ∼201 taxa). Ultra large datasets with thousands of taxa need to be analyzed, especially to demonstrate the power of ML based techniques in leveraging the latent features of phylogenetic data. Although we have appropriately adapted the MF and AE based techniques for imputing distance matrices, further parameter tuning and customization in the underlying deep learning architecture may improve the performance of our proposed techniques. We leave these as future works.

## Conclusions

In this study, we have presented two imputation techniques, inspired from matrix factorization and deep learning architecture, to reconstruct phylogenetic trees from partial distance matrices. Experimental results on both simulated and real biological datasets show that our methods match or improve upon the alternate best techniques under various model conditions with varying numbers of taxa, sequence lengths, and amounts of gene tree discordance. We also evaluated these methods using different DNA sequence evolution models and missingness mechanisms.

Estimating phylogenetic trees in the presence of missing data is sufficiently complex and hence existing methods cannot fully comprehend or predict the relationships among the taxa from partial distance matrices. Thus, the goal here should be the creation of an appropriate model to capture the underlying data distribution; the model should account for as much phylogenetic data as possible to impute the missing entries. This view emphasizes the importance of ML for distance matrix imputation. Moreover, we aimed to develop appropriate unsupervised models. Unsupervised learning approaches have advantages over supervised methods particularly when the data are heterogeneous, which are often so with various phylogenetic dataset and therefore the supervised models trained on distance matrices on a particular set of taxa may not be generalizable to a new set of taxa.

We have shown that MF and AE are robust, and can handle high amounts of missing data. Unlike other methods [36], MF and AE do not require the molecular clock assumption. Moreover, deep learning based methods (e.g., autoencoders) are able to automatically learn latent representations and complex inter-variable associations, which is not possible for heuristic based methods. Therefore, this study lays a firm and broad foundation for applying ML based techniques in various problems in phylogenomics. Considering the rapidly increasing amount of phylogenomic datasets, and the prevalence of accompanying missing data, the timing of our proposed approaches seems appropriate. We believe that the proposed imputation techniques represent a major step towards solving real world instances in phylogenomics.

## Methods

### Matrix factorization (MF)

Matrix factorization (MF) has become popular since 2006, when one group of competitors for the Netflix Prize that year used this technique [53, 75]. This method is usually being applied in recommender systems [76], and is used to discover latent features between two interacting entities. Matrix factorization is a class of collaborative filtering algorithms [77], which predicts users’ future interest by analyzing their past behavior.

Intuitively, there should be some latent features behind how a certain user rates an item. For example, movie ratings by users generally rely on many features, including genre, actors, etc. If a certain individual gives high ratings to action movies, we can expect him to do the same to another action movie which is not yet rated by him. Discovering the latent features will thus help predict users’ future preferences.

Matrix Factorization has previously been used in imputing missing data in various domains of bioinformatics, including analyzing scRNA-seq with missing data [78], handling missing data in genome-wide association studies (GWAS) [79], and identifying cancerous genes [80]. In this study, we have adapted this idea for imputing missing entries in a distance matrix for phylogenetic estimation. If the distance between two taxa *A* and *B* is not known, we can predict the distance by analyzing their distances with other taxa using the concept of matrix factorization (with appropriate customization).

Let *S* be a set of *N* OTUs (operational taxonomic units). Let *R* be an |*N*|×|*N*| distance matrix comprising the distances between any two OTUs. If we want to find *K* latent features of distances, we need to find two matrices *X* and *Y*, where the dimensions of *X* and *Y* are |*N*|×*K*. We used *K*=*N* in our implementation. The product of *X* and *Y*^*T*^ will then approximate *R* as follows.

2 $$ R \approx X\times Y^{T} = \hat{R}  $$

However, as matrix *R* (and $\hat {R}$) are symmetric, meaning that *r*_*ij*_=*r*_*ji*_ (and $\hat {r}_{ij} = \hat {r}_{ji}$), we only consider the lower triangular portion of the matrix. We impute a distance $\hat {r}_{ij}$ between two OTUs as follows.

3 $$ \hat{r}_{ij} = \sum_{k=1}^{K} x_{ik}y_{kj}  $$

We randomly initialize *X* and *Y* and try to determine the error between *R* and the product of *X* and *Y*. Then we iteratively update *X* and *Y* so that the error is reduced. We considered the squared error as the errors can be both positive and negative. We used a regularization parameter *β* to avoid overfitting. Thus, we calculate the error as follows.

4 $$  \begin{aligned} e_{ij}^{2} &= (r_{ij} - \hat{r}_{ij})^{2} + \frac{\beta}{2}\sum_{k=1}^{K}(||X||^{2} + ||Y||^{2}) \\ &= (r_{ij} - \sum_{k=1}^{K} x_{ik}y_{kj})^{2} + \frac{\beta}{2}\sum_{k=1}^{K}(||X||^{2} + ||Y||^{2}) \end{aligned}  $$

In order to minimize the error defined in Eqn. 4, the directions for modifying *x*_*ik*_ and *y*_*kj*_ need to be identified. This means we need to find the gradient at current values, which we do by differentiating Eqn. 4 with respect to *x*_*ik*_ and *y*_*kj*_ separately. Thus, we use the following update rules.

5 $$ {{}\begin{aligned} x_{ik} (updated) &= x_{ik} + \alpha \frac{\partial}{\partial x_{ik}} e_{ij}^{2} &= x_{ik} + \alpha(2e_{ij}y_{kj} - \beta x_{ik}) \end{aligned}}   $$

6 $$ {{}\begin{aligned} y_{kj} (updated) &= y_{kj} + \alpha \frac{\partial}{\partial y_{kj}} e_{ij}^{2} &= y_{kj} + \alpha(2e_{ij}x_{ik} - \beta y_{kj}) \end{aligned}}   $$

In Eqns. 5 and 6, *α* is a constant which determines the rate to approach the minimum error. We experimented with a range of values (10^−4^∼10^−1^) of *α* and *β* from the implementations in [81], and set *α* = 0.002 and *β* = 0.02 as these values provided reliable performance. However, further parameter tuning may improve both the accuracy and convergence time. We perform these steps iteratively until the total error *E* ($= \sum e_{ij}$) converges to a pre-specified threshold value (10^−6^) or 10,000 iterations take place. Algorithm 1 shows our MF-based imputation process.


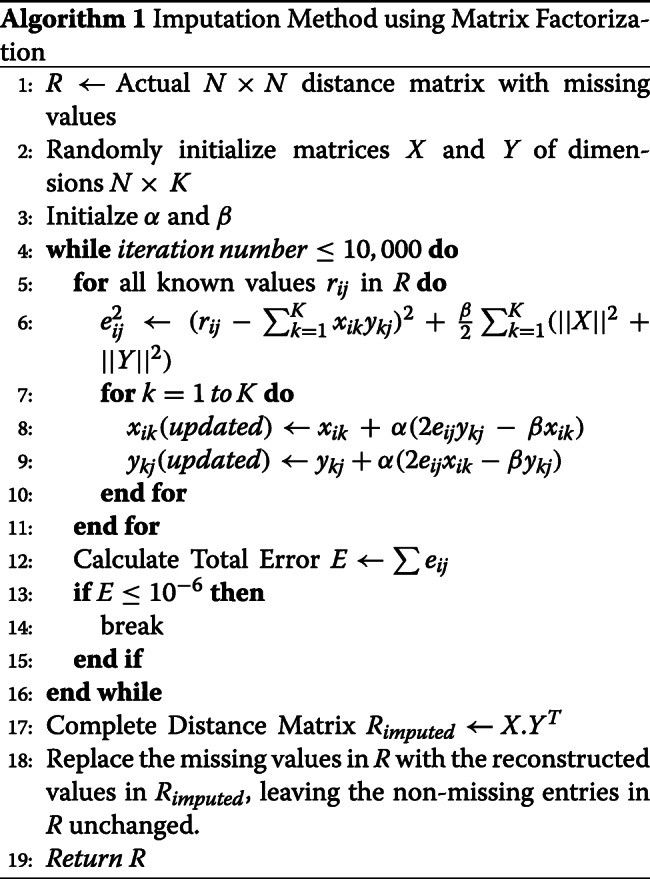


### Autoencoder (AE)

**Fig. 3:**
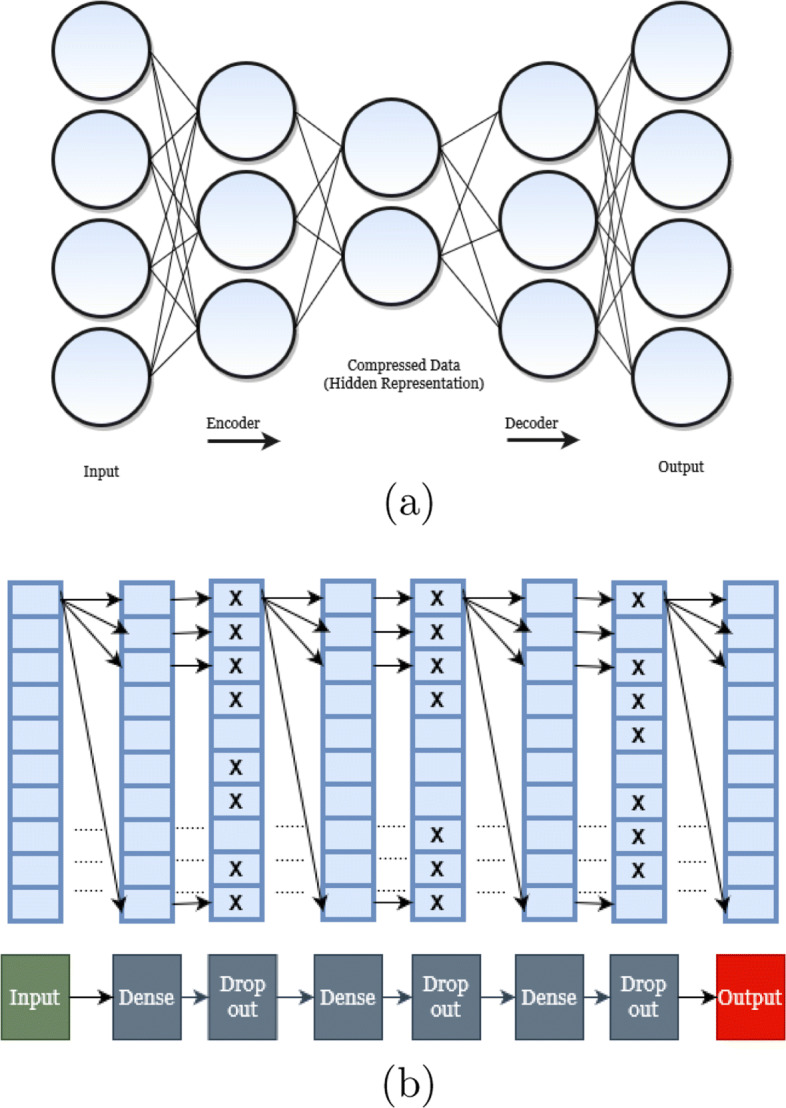
**a** General overview of an autoencoder. **b** A schematic of our proposed autoencoder model. The *X*’s in the dropout layers symbolically denote that their weights will be set to zero

An autoencoder (AE) is a type of artificial neural network that learns to copy its input to its output. This is achieved by learning efficient data codings in an unsupervised manner to recreate the input. An autoencoder first compresses the input into a latent space representation and then reconstructs the output from that representation. It tries to learn a function *g*(*f*(*x*))≈*x*, where *f*(*x*) encodes the input *x* and *g*(*f*(*x*)) reconstructs the input *x* using decoder. Figure 3a shows a general overview of autoencoders.

Autoencoders have been used in integrative analyses of biomedical big data. Its ability to reduce the dimension and extract non-linear features [82] have been leveraged by many studies. In one oncology study, autoencoders have been able to extract cellular features, which can correlate with drug sensitivity involved with cancer cell lines [83]. An autoencoder was also used to discover two liver cancer sub-types that had distinguishable chances of survival [84]. Moreover, some recent successful data imputation methods have been developed based on autoencoders [85–87]. Autoimpute [85] can be an example which imputes single cell RNA-seq gene expression data. Autoencoder-based methods such as [86] and [87] have surpassed older ML techniques on various real life datasets.

In this study, we developed an *undercomplete* autoencoder [54] to predict the missing values in a distance matrix. The goal of an undercomplete autoencoder is to learn the most salient features of data by putting a constraint on the amount of information that can flow through the network. We do not need any regularization term here because an undercomplete autoencoder maximizes the probability of data rather than copying the input to the output.

Our architecture has been inspired by an open source library, *FancyImpute* [88], which is a library for imputation algorithms and is implemented in Python language. Our model has 3 hidden layers with ReLU (Rectified Linear Unit) *activation functions* [89]. The *dropout rate* is set to 0.75, which appears to work better than other values. A sigmoid function [90] is used as the activation function for output layer. FancyImpute iteratively updates the imputed values where a prediction from the previous iteration is updated according to Eq. 7. We used the default predefined weights from the FancyImpute library.

7 $$ x^{\prime} = (1 - w)x + wp   $$

In Eq. 7, *x*^′^ = updated value, *x* = old value, *w* = predefined weight, and *p* = predicted value from the autoencoder.


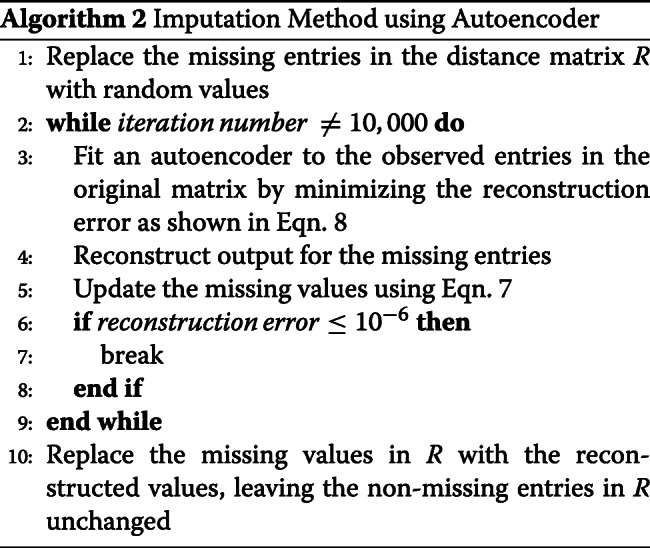


Our model takes as input a distance matrix *R* with missing entries. First, the missing values in *R* are replaced with random values. Next, using the architecture shown in Fig. 3b, our model tries to fit the input (*R*) to output (*R*^′^). It tries to progressively improve the prediction by minimizing the reconstruction error (loss function) where the error is computed based on the non-missing entries of the original matrix. We have used the mean squared error (MSE) as the reconstruction error function *L*(*R*,*R*^′^), which minimizes the difference between the input *R* and the autoencoder’s output *R*^′^ considering only the non-missing entries. Let $\mathcal {NM}$ be the set of non-missing entries in *R*. Then, *L*(*R*,*R*^′^) is computed as follows.

8 $$  L(R, R^{\prime}) = \sum_{i \in \mathcal{NM}}|R_{i} - R_{i}^{\prime} |^{2}.  $$

We replace the missing entries with the imputed values and keep the original non-missing values unchanged once a certain number of iterations (10,000) have taken place or the reconstruction error has gone below a pre-specified threshold value (10^−6^). Algorithm 2 shows our AE-based imputation process.

### Software implementation

The proposed methods have been developed in Python 3.5 using various libraries, namely, *easygui*, *pandas*, *numpy*, *matplotlib*, *seaborn*, *tensorflow*, and *keras*. The methods have been developed as cross-platform applications.

The 201-taxon dataset is available at https://sites.google.com/eng.ucsd.edu/datasets/astral/astral-ii[72].

The 37-taxon dataset is available at https://sites.google.com/eng.ucsd.edu/datasets/binning [67].

The 24-taxon dataset is available at 10.7717/peerj.5321/supp-1 [47].

The 10-, 9-, and 7-taxon datasets are available in the DAMBE software package (http://dambe.bio.uottawa.ca/DAMBE/dambe.aspx) [56].

## Data Availability

The proposed methods are available in open source form at https://github.com/Ananya-Bhattacharjee/ImputeDistances. All the datasets analyzed in this paper are from previously published studies and are publicly available.

## Abbreviations

AE: Autoencoder
GWAS: Genome-wide association study
ILS: Incomplete lineage sorting
MCMC: Markov chain Monte Carlo
MF: Matrix factorization
ML: Machine learning
MSE: Mean-squared error
NJ: Neighbor joining
OTU: Operational taxonomic unit
ReLU: Rectified Linear Unit
RF: Robinson Foulds
scRNA-seq: Single-cell RNA sequencing
SNP: Single nucleotide polymorphism
UPGMA: Unweighted pair group method with arithmetic mean
